# Automatic MRI volumetry in asymptomatic cases at risk for normal pressure hydrocephalus

**DOI:** 10.3389/fnagi.2023.1242158

**Published:** 2023-11-03

**Authors:** Sven Haller, Marie-Louise Montandon, Cristelle Rodriguez, François R. Herrmann, Panteleimon Giannakopoulos

**Affiliations:** ^1^CIMC - Centre d’Imagerie Médicale de Cornavin, Geneva, Switzerland; ^2^Department of Surgical Sciences, Radiology, Uppsala University, Uppsala, Sweden; ^3^Faculty of Medicine, University of Geneva, Geneva, Switzerland; ^4^Department of Radiology, Beijing Tiantan Hospital, Capital Medical University, Beijing, China; ^5^Department of Rehabilitation and Geriatrics, Geneva University Hospitals and University of Geneva, Geneva, Switzerland; ^6^Division of Institutional Measures, Medical Direction, Geneva University Hospitals, Geneva, Switzerland; ^7^Department of Psychiatry, Faculty of Medicine, University of Geneva, Geneva, Switzerland

**Keywords:** Alzheimer, brain aging, hippocampus, MRI markers, normal pressure hydrocephalus, vector machine assessment, white matter

## Abstract

The occurrence of significant Alzheimer’s disease (AD) pathology was described in approximately 30% of normal pressure hydrocephalus (NPH) cases, leading to the distinction between neurodegenerative and idiopathic forms of this disorder. Whether or not there is a specific MRI signature of NPH remains a matter of debate. The present study focuses on asymptomatic cases at risk for NPH as defined with automatic machine learning tools and combines automatic MRI assessment of cortical and white matter volumetry, risk of AD (AD-RAI), and brain age gap estimation (BrainAge). Our hypothesis was that brain aging and AD process-independent volumetric changes occur in asymptomatic NPH-positive cases. We explored the volumetric changes in normal aging-sensitive (entorhinal cortex and parahippocampal gyrus/PHG) and AD-signature areas (hippocampus), four control cortical areas (frontal, parietal, occipital, and temporal), and cerebral and cerebellar white matter in 30 asymptomatic cases at risk for NPH (NPH probability >30) compared to 30 NPH-negative cases (NPH probability <5) with preserved cognition. In univariate regression models, NPH positivity was associated with decreased volumes in the hippocampus, parahippocampal gyrus (PHG), and entorhinal cortex bilaterally. The strongest negative association was found in the left hippocampus that persisted when adjusting for AD-RAI and Brain Age values. A combined model including the three parameters explained 36.5% of the variance, left hippocampal volumes, and BrainAge values, which remained independent predictors of the NPH status. Bilateral PHG and entorhinal cortex volumes were negatively associated with NPH-positive status in univariate models but this relationship did not persist when adjusting for BrainAge, the latter remaining the only predictor of the NPH status. We also found a negative association between bilateral cerebral and cerebellar white matter volumes and NPH status that persisted after controlling for AD-RAI or Brain Age values, explaining between 50 and 65% of its variance. These observations support the idea that in cases at risk for NPH, as defined by support vector machine assessment of NPH-related MRI markers, brain aging-related and brain aging and AD-independent volumetric changes coexist. The latter concerns volume loss in restricted hippocampal and white matter areas that could be considered as the MRI signature of idiopathic forms of NPH.

## 1. Introduction

Normal pressure hydrocephalus (NPH) is a brain disorder characterized by an increase in cerebrospinal fluid (CSF) volume in the subarachnoid and ventricular spaces that, in its clinical phases, leads to gait disturbance, urinary incontinence, and dementia (Hakim and Adams, 1965; Jaraj et al., 2014). The relationship between AD pathology and NPH status is quite complex. Müller-Schmitz and colleagues reported that the presence of positive CSF-AD biomarkers was associated with better prognosis after shunting and proposed a distinction between neurodegenerative and idiopathic forms of NPH (Muller-Schmitz et al., 2020). Along the same line, clinically overt NPH with better response to shunting is accompanied by significant AD histopathological changes in 19–30% of cases (Pomeraniec et al., 2016; Graff-Radford and Jones, 2019). For others, the frequent comorbidity between AD pathological changes and NPH status may worsen the prognosis of the latter (Allali et al., 2018). In particular, aggregations of AD-related proteins (amyloid-β and hyperphosphorylated tau) in frontal cortical biopsies were associated with a poor shunt response in cases with suspected NPH (Kojoukhova et al., 2017), but the inverse association or negative data were also reported (Luikku et al., 2016; Pomeraniec et al., 2016). Recently, a hydrocephalic variant of AD was described and characterized by better memory and language performance compared to typical AD, but no significant improvement after shunt surgery (Jang et al., 2022).

Several MRI markers have been proposed to identify NPH-positive cases in elderly cohorts. Early studies proposed traditional 2-dimensional linear measures such as Evan’s, bicaudate index, and callosal angle (Marmarou et al., 2005; Ishikawa et al., 2008). The disproportional enlargement of the ventricles and Sylvian fissure and the tight high convexity in posterior callosomarginal fissures were also used with good levels of accuracy for the distinction between NPH and healthy controls (Hashimoto et al., 2010; Yamada et al., 2017). However, some volumetric studies demonstrating z-axial ventricular expansion indicated that these morphological indices may be insufficient to fully describe the patterns of ventricular enlargement versus brain atrophy in mixed cohorts of elderly individuals with AD pathological changes (Ambarki et al., 2010; Toma et al., 2011). With respect to the NPH/AD distinction, the use of three-dimensional linear measures and diffusion tensor imaging-based assessment of splenial angle displayed good to excellent levels of accuracy (Chan et al., 2021; Soon et al., 2021).

Previous studies using automatic machine learning tools focused on the distinction between NPH patients, AD, and other forms of dementia and healthy controls (Serulle et al., 2014; Miskin et al., 2017; Gunter et al., 2019; Duan et al., 2020). Volumetric MRI predictors of gray and white matter reached an accuracy close to 95% in differentiating NPH/AD from controls (Miskin et al., 2017). In an automatic MRI brain segmentation study, the presence of high ventricular volumes and preserved gray matter volumes allowed for a highly accurate distinction between shunt-responsive NPH patients and AD cases (Serulle et al., 2014). More recently, Rau et al. (2021) proposed a new support vector machine (SVM) algorithm processing 3D T1-weighted datasets which were routinely acquired in NPH patients and demonstrated highly reliable detection of NPH patterns (compared to human readings).

The development of NPH is a long process in old age, and radiographic features show volumetric changes 3 or more years before the onset of clinical symptoms (Engel et al., 2018). As the radiological features may precede clinical symptoms, the automatic detection during the asymptomatic stage could be an alternative in order to prevent dementia and associated costs in older individuals (Siraj, 2011; Engel et al., 2018; Rau et al., 2021). In the context of a large community-based prospective study on healthy aging, we identified a series of asymptomatic, radiologically defined cases at risk for NPH according to the algorithm proposed by Rau et al. (2021). Of note, previous studies showed that asymptomatic ventriculomegaly with features of idiopathic normal pressure hydrocephalus progresses to clinically overt NPH in 10–20% of cases per year (Iseki et al., 2009; Kimihira et al., 2020). The primary objective of this study was to explore whether there were MRI predictors of the radiological NPH-positive status independent of brain aging and AD-related volumetric changes in old age. Our hypothesis was that brain aging and AD process-independent volumetric changes occur in asymptomatic NPH-positive patients and may help to define the idiopathic dimension of this disorder. After controlling for brain aging and AD status using automatic voxel-based support-vector-machine (SVM)-learning MRI parameters, we explored the volumetric changes in normal aging-sensitive (entorhinal cortex and parahippocampal gyrus/PHG) and AD-signature areas (hippocampus), four control cortical areas (frontal, parietal, occipital, and temporal) but also in the cerebral and cerebellar white matter given the well-known sensitivity of subcortical white matter in NPH (Kristensen et al., 1996; Momjian et al., 2004; Bradley, 2016).

## 2. Methods

### 2.1. Participants

The study was approved by the local Ethics Committee, and all participants gave written informed consent prior to inclusion. The selection of cases among participants of a still ongoing cohort study aiming to identify predictive biomarkers of subtle cognitive decline among healthy elders was described in detail elsewhere (Montandon et al., 2020a). Briefly, the present cohort included only healthy controls with preserved cognition; no history of psychiatric, neurological, and major medical conditions; and no regular use of psychotropic medication (Zanchi et al., 2017; Montandon et al., 2020a,b; Giannakopoulos et al., 2020, 2022b). All cases were recruited via advertisements in local newspapers and media. The cohort of reference included 526 elderly Caucasian white individuals living in the Geneva and Lausanne catchment area. Due to the need for excellent French knowledge (to participate in detailed neuropsychological testing), most of the participants were Swiss (or born in French-speaking European countries, 92%). Substantial vascular burden as evidenced by subtle cardiovascular symptoms, hypertension (non-treated), and a history of stroke or transient ischemic episodes was an additional exclusion criterion. All cases included in this cohort had three neuropsychological evaluations (baseline, 18 months and 54 months), structural brain MRI at baseline, APOE genotyping, and amyloid and FDG PET at the last follow-up. For the purpose of this study, we focused on baseline MRI data.

### 2.2. Neuropsychological assessment

At baseline, all individuals were evaluated with an extensive neuropsychological battery, including the Mini-Mental State Examination (MMSE; Folstein et al., 1975), the Hospital Anxiety and Depression Scale (HAD; Zigmond and Snaith, 1983), and the Lawton Instrumental Activities of Daily Living (IADL; Barberger-Gateau et al., 1992). The cognitive assessment included (a) attention (Digit-Symbol-Coding; Wechsler, 1997) and Trail Making Test A (Reitan, 1958); (b) working memory (verbal: Digit Span Forward; Wechsler, 1955) and visuo-spatial: Visual Memory Span (Corsi; Milner, 1971); (c) episodic memory (verbal: RI-48 Cued Recall Test; Buschke et al., 1997) and visual: Shapes Test (Baddley et al., 1994); (d) executive functions (Trail Making Test B; Reitan, 1958), Wisconsin Card Sorting Test, and Phonemic Verbal Fluency Test (Heaton et al., 1993); (e) language (Boston Naming; Kaplan et al., 1983); (f) visual gnosis (Ghent Overlapping Figures); (g) praxis: ideomotor (Schnider et al., 1997), reflexive (Poeck, 1985), and constructional (Consortium to Establish a Registry for Alzheimer’s Disease [CERAD] and Figures copy; Welsh et al., 1994). All individuals were also evaluated with the Clinical Dementia Rating Scale (CDR; Hughes et al., 1982). In agreement with the criteria of Petersen et al. (2001), participants with a CDR of 0.5 but no dementia and a score exceeding 1.5 standard deviations below the age-appropriate mean in any of the cognitive tests were classified as MCI and were excluded. Participants with neither dementia nor MCI were classified as cognitively healthy controls. As part of the longitudinal arm of our cohort investigation, all of these cases underwent a 7-year follow-up with three additional assessments using the same neuropsychological battery in order to identify the very first stages of cognitive decrement in healthy aging. Only baseline data were used for the purposes of the present study.

### 2.3. MR imaging

Imaging data were acquired on a 3 T MRI scanner (TRIO SIEMENS Medical Systems, Erlangen, Germany). The structural high-resolution T1-weighted anatomical scan was performed with the following fundamental parameters: 256 × 256 matrix, 176 slices, 1 mm isotropic, TR = 2.27 ms. Additional sequences (T2w imaging, susceptibility-weighted imaging, and diffusion tensor imaging) were used to exclude incidental brain lesions.

### 2.4. Image pre-processing

The structural T1-images were segmented into grey matter, white matter, and cerebro-spinal fluid (CSF) using SPM12 (Welcome Trust Center for Neuroimaging, warped into the MNI [Montreal Neurological Institute]) space (using modulation of grey value by the Jacobian of the warp) and smoothed by full width-half-max 3 mm filter, like what is done for usual voxel-based morphometry analyses. The segmentation results were inspected visually prior to further processing, and none of the cases had to be excluded because of obvious failures. Mesial temporal atrophy (MTA) was assessed according to the established score (Scheltens et al., 1995), ranging from zero (no atrophy) to four (significant atrophy).

### 2.5. Automatic assessment of NPH scores

The definition of the final sample was made on the basis of the automatic assessment of NPH scores as described in Rau et al. (2021). Briefly, SVM was trained in NPH patients treated with ventriculo-peritoneal shunts and healthy controls, with the smoothed segmentations of gray matter and CSF as inputs. The output is an NPH probability score between 0 and 100. We screened all of the cases included in our cohort of reference and identified 30 cases with NPH scores superior to 30 corresponding to intermediate and high probability of being an asymptomatic NPH-positive case. Subsequently, we randomly selected 30 cases with NPH scores lower than five corresponding to NPH-negative cases. These 60 cases were considered for further MRI analyses.

### 2.6. Automatic assessment of BrainAGE

BrainAGE was estimated according to the approach described previously by Franke et al. (2010) and Giannakopoulos et al. (2022a). Briefly, the BrainAGE framework utilized relevance vector regression (RVR) using a linear kernel and a linear combination of pre-processed GM and WM images (Tipping, 2000, 2001). We used a modified approach to our pre-processing as described previously [25]. T1-weighted images were pre-processed using the CAT12 toolbox^1^ and the SPM12 software,^2^ running under MATLAB.^3^ To train the age estimation framework, we used MRI data of 547 healthy subjects (242 men) from the publicly accessible IXI cohort,^4^ aged 19–86 years (mean (SD) = 48.1 (16.6) years). In brief, the BrainAGE framework utilizes relevance vector regression (RVR) using a linear kernel and a linear combination of pre-processed GM and WM images. The difference between estimated and chronological age yields the individual brain age gap estimation (BrainAGE) score, with positive values indicating accelerated and negative values indicating decelerated structural brain aging. Recent work has demonstrated that this method provides reliable and stable estimates of BrainAGE at a mean absolute error of 3.322 years, rendering this framework at least equal to several recently introduced deep learning algorithms (Franke et al., 2010).

### 2.7. Automatic assessment of AD-RAI

An automated assessment of the AD-RAI was performed using a voxel-based SVM. The basic principle is described in Kloppel et al. (2008). The smoothed segmentations of grey matter and CSF are used as direct inputs to an SVM. In the context of this work, a customized research version of the “VEOmorph” Software^5^ was trained with data from another study (in total, 741 subjects, 445 Healthy Controls, 234 AD, 39 FTLD, and 23 LB). The age distribution in the training (range 45–90 years and median value 73 ± 8.2 years) covers the range of the population. In the present study, Ground-truth labeling was based on clinical diagnoses. Fivefold cross-validation was performed. The AD-RAI was determined as the probability score of the SVM output as a value between zero and one. AD-RAI score was calculated as described in detail before. The AD-RAI was determined as the probability score of the SVM output as a value between zero and one. The trained algorithm was applied to the current dataset without any further adjustments as in our previous work (Giannakopoulos et al., 2022a).

### 2.8. Automatic MR volumetry

Automatic MR volumetry was performed with the fully automated multi-atlas segmentation tool cNeuro (Combinostics Ltd., Tampere, Finland)^6^ as described by Lotjonen et al. (2010). The regions of interest for automatic volumetry included three early AD-signature areas (hippocampus, parahippocampal gyrus, and entorhinal cortex), three control areas (occipital, parietal, and frontal lobes) bilaterally, and cerebral and cerebellum white matter.

## 3. Results

**Figure 1:**
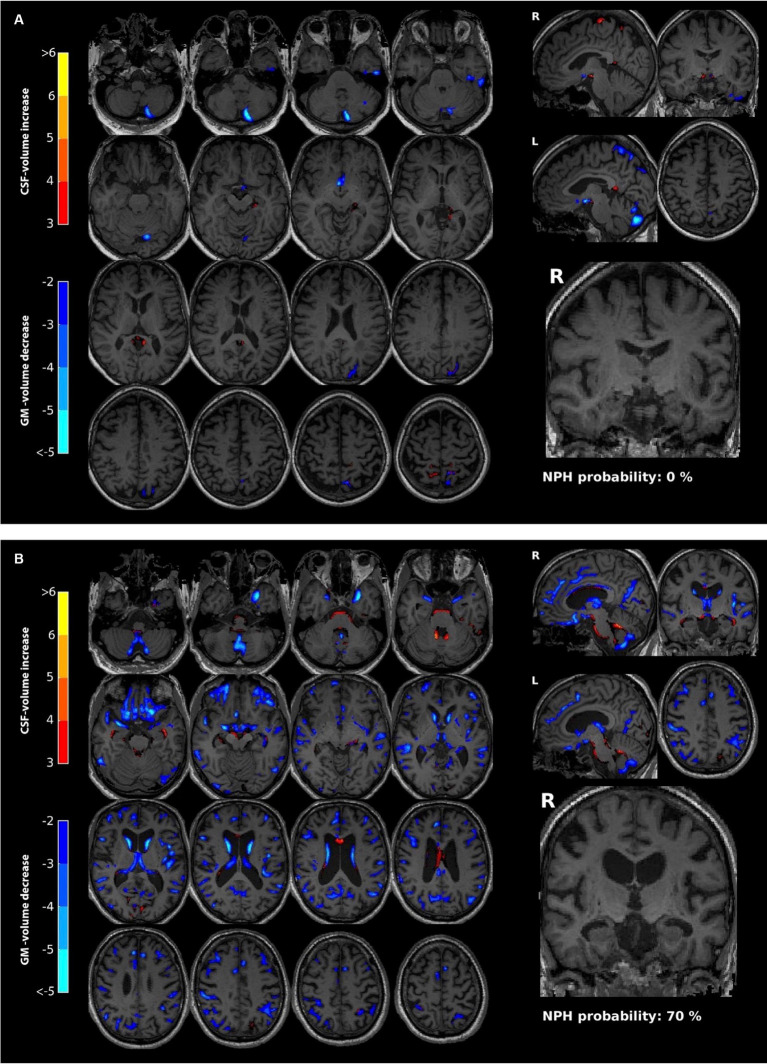
Illustration of a 78-year-old man **(A)**. The left panel (axial) and top right panel (sagittal) show a voxel-wise comparison of this individual to an age- and gender-matched control cohort. As there is no significant atrophy, only a few very small clusters (in blue) indicate a minimal decrease in GM. The coronal slice at the level of the hippocampus (bottom right) shows no significant atrophy of the hippocampus (MTA score 0). There is no significant NPH configuration indicated by an NPH probability of 0%. A case of a 75-year-old man **(B)** shows several features of NPH configuration including a prominent ventricular system, slight crowding of the sulci of the vertex, minimal posterior cingulate sulcus sign, and minimal dilation of the sylvian fissure. Moreover, several regions show a reduction in GM, indicated by the blue voxels. The NPH configuration probability is estimated at 70%. Note that dilation of the temporal horns is a feature of NPH configuration, which might lead to an overestimation of the MTA score. In the current example, the MTA score is one.

Demographic and clinical data of the present series are summarized in Table 1. Group comparisons showed higher MTA scores as well as BrainAge and AD-RAI values in NPH > 30 compared to NPH < 5 groups. In addition, MRI volumes in both AD-signature areas and cerebral and cerebellum white matter were significantly lower in NPH > 30 than in NPH < 5 groups. With one exception (PHG right), all of these differences persisted after Benjamini-Hochberg correction for multiple comparisons (Table 1; Figure 1).

**Table 1:** Demographic, clinical, and MRI characteristics in the present series.

A	NPH < 5	NPH > 30	Total	*p* value	BH
*N*	30	30	60	1.0000	
Age	73.633 ± 3.681	73.600 ± 3.607	73.617 ± 3.613	0.9719	
MMSE score	28.867 ± 1.008	28.233 ± 1.223	28.550 ± 1.156	0.1404	
Female sex	13 (43.3%)	13 (43.3%)	26 (43.3%)	1.0000	
Education				0.0931	
<12 years	8 (26.7%)	3 (10.0%)	11 (18.3%)		
12 years	7 (23.3%)	14 (46.7%)	21 (35.0%)		
>12 years	15 (50.0%)	13 (43.3%)	28 (46.7%)		
Fazekas score				0.7477	
Absent	11 (36.7%)	10 (33.3%)	21 (35.0%)		
Mild	15 (50.0%)	14 (46.7%)	29 (48.3%)		
Moderate	4 (13.3%)	4 (13.3%)	8 (13.3%)		
Severe	0 (0.0%)	2 (6.7%)	2 (3.3%)		
Number of microbleeds				0.2536	
0	25 (83.3%)	19 (63.3%)	44 (73.3%)		
1	3 (10.0%)	7 (23.3%)	10 (16.7%)		
2	2 (6.7%)	2 (6.7%)	4 (6.7%)		
3	0 (0.0%)	2 (6.7%)	2 (3.3%)		
Mesial temporal atrophy (MTA)				0.0005	*
0	13 (43.3%)	2 (6.7%)	15 (25.0%)		
1	15 (50.0%)	17 (56.7%)	32 (53.3%)		
2	2 (6.7%)	11 (36.7%)	13 (21.7%)		
AD-RAI	0.066 ± 0.105	0.152 ± 0.173	0.109 ± 0.149	0.0238	*
BrainAGE	−0.700 ± 3.309	2.937 ± 2.231	1.056 ± 3.360	<0.0001	*
**MRI volumes**					
Hippocampus right	3.750 ± 0.316	3.438 ± 0.305	3.594 ± 0.346	0.0003	*
Hippocampus left	3.562 ± 0.263	3.227 ± 0.236	3.394 ± 0.300	<0.0001	*
Entorhinal cortex right	2.360 ± 0.268	2.205 ± 0.220	2.282 ± 0.255	0.0173	*
Entorhinal cortex left	2.240 ± 0.195	2.107 ± 0.231	2.173 ± 0.222	0.0185	*
Parahippocampal gyrus right	2.943 ± 0.224	2.825 ± 0.200	2.884 ± 0.219	0.0363	
Parahippocampal gyrus left	3.219 ± 0.330	2.998 ± 0.210	3.108 ± 0.296	0.0032	*
Cerebral white matter right	193.947 ± 10.478	181.022 ± 10.515	187.485 ± 12.279	<0.0001	*
Cerebral white matter left	191.588 ± 10.316	179.879 ± 10.541	185.734 ± 11.907	0.0001	*
Cerebellum white matter right	12.059 ± 1.232	9.989 ± 0.948	11.024 ± 1.509	<0.0001	*
Cerebellum white matter left	12.627 ± 1.341	10.886 ± 0.950	11.757 ± 1.448	<0.0001	*

BH, Benjamini-Hochberg; *p* threshold (0.0286); *statistically significant.

In univariate regression models and among brain aging and AD-signature areas, NPH status was associated with decreased volumes in the hippocampus, PHG, and entorhinal cortex bilaterally (Tables 2, 3). The strongest negative association was found in the left hippocampus with a 27.6% explained variance for this single parameter. This association persisted when adjusting for AD-RAI and Brain Age values. A combined model including the three parameters explained 36.5% of the variance, left hippocampal volumes, and BrainAge values, which remained independent predictors of the NPH status. Similar observations were found in the right hippocampus but in multivariable models; only Brain Age remained a significant predictor of the NPH status (33.2% of the variance, Table 2). A different pattern of associations was observed with respect to PHG and entorhinal cortex volumes. Bilateral PHG volumes were negatively associated with NPH-positive status in univariate models, but this relationship did not persist when adjusting for BrainAge values that remained the only predictor of the NPH status, accounting for more than 30% of its variance. The same was true for entorhinal cortex volumes (Table 3).

**Table 2:** Prediction of NPH status (NPH < 5 vs. NPH > 30) with univariate and multiple logistic regression models.

	Left		Right	
MRI volume	OR (95%CI)	*p*	OR (95%CI)	*p*
Hippocampus	0.00 (0.00, 0.07)	0.0003	0.04 (0.00, 0.28)	0.0014
Hippocampus	0.00 (0.00, 0.11)	0.0008	0.04 (0.01, 0.32)	0.0022
AD index	17.96 (0.12, 2606.09)	0.2555	124.86 (0.79, 19690.07)	0.0616
Hippocampus	0.02 (0.00, 0.60)	0.0237	0.16 (0.02, 1.60)	0.1192
BrainAGE	1.49 (1.07, 2.09)	0.0194	1.64 (1.18, 2.28)	0.0035
Hippocampus	0.02 (0.00, 0.69)	0.0292	0.14 (0.01, 1.38)	0.0918
AD index	9.13 (0.04, 2337.68)	0.4345	32.82 (0.13, 8561.53)	0.2188
BrainAGE	1.47 (1.04, 2.07)	0.0292	1.57 (1.12, 2.21)	0.0092

**Table 3:** Prediction of NPH status (NPH < 5 vs. NPH > 30) with univariate and multiple logistic regression models.

	Left		Right	
MRI volume	OR (95%CI)	*p*	OR (95%CI)	*p*
Entorhinal	0.05 (0.00, 0.68)	0.0252	0.07 (0.01, 0.70)	0.0242
Entorhinal	0.06 (0.00, 0.88)	0.0398	0.07 (0.01, 0.80)	0.0330
AD-RAI	109.47 (0.86, 13879.13)	0.0574	120.07 (1.10, 13082.31)	0.0454
Entorhinal	0.16 (0.01, 3.34)	0.2399	0.25 (0.01, 4.49)	0.3444
BrainAGE	1.67 (1.22, 2.29)	0.0015	1.70 (1.24, 2.33)	0.0011
Entorhinal	0.16 (0.01, 3.24)	0.2320	0.23 (0.01, 4.49)	0.3343
AD-RAI	20.77 (0.08, 5274.94)	0.2830	18.49 (0.09, 3665.80)	0.2798
BrainAGE	1.62 (1.17, 2.23)	0.0035	1.65 (1.19, 2.28)	0.0026
PHG	0.04 (0.00, 0.42)	0.0070	0.07 (0.00, 0.93)	0.0443
PHG	0.04 (0.00, 0.42)	0.0082	0.09 (0.01, 1.36)	0.0819
AD-RAI	132.83 (1.21, 14630.23)	0.0415	75.21 (0.84, 6764.10)	0.0598
PHG	0.16 (0.01, 2.49)	0.1913	0.24 (0.01, 5.04)	0.3573
BrainAGE	1.63 (1.19, 2.25)	0.0027	1.71 (1.25, 2.34)	0.0009
PHG	0.14 (0.01, 2.31)	0.1709	0.28 (0.01, 5.75)	0.4090
AD-RAI	21.08 (0.10, 4235.97)	0.2599	14.50 (0.07, 2870.12)	0.3215
BrainAGE	1.57 (1.12, 2.18)	0.0080	1.67 (1.21, 2.30)	0.0019

PHG, parahippocampal gyrus.

Among control areas (frontal, occipital, and parietal lobes), there were no significant associations between the volumes and NPH status (data not shown). In contrast, we found a strong negative association between cerebral white matter volumes bilaterally and increased NPH values (35.6% of variance in the left and 37.8% in the right hemisphere) that persisted after adjustment for AD-RAI or Brain Age values (Table 4). In a combined model including the three MRI parameters, cerebral white matter volumes and Brain Age remained significant predictors of the NPH status and explained 50.1 and 65% of its variance. Similar data were obtained with respect to cerebellum white matter volume bilaterally (32.2% of variance in the left and 35.4% in the right hemisphere). In a combined model, both cerebellar white matter volumes and Brain Age values were independent predictors of the NPH positivity explaining between 49.2% (left) and 62% (right) of its variance (Table 4).

**Table 4:** Prediction of NPH status (NPH < 5 vs. NPH > 30) with univariate and multiple logistic regression models.

	Left		Right	
MRI volume	OR (95%CI)	*p*	OR (95%CI)	*p*
Cerebral white matter	0.89 (0.84, 0.95)	0.0007	0.89 (0.83, 0.95)	0.0004
Cerebral white matter	0.90 (0.84, 0.96)	0.0010	0.89 (0.84, 0.95)	0.0006
AD-RAI	95.61 (0.75, 12221.24)	0.0654	53.02 (0.42, 6669.73)	0.1075
Cerebral white matter	0.87 (0.80, 0.95)	0.0024	0.87 (0.80, 0.95)	0.0017
BrainAGE	1.96 (1.29, 3.00)	0.0018	1.92 (1.25, 2.93)	0.0028
Cerebral white matter	0.87 (0.80, 0.95)	0.0027	0.87 (0.80, 0.95)	0.0020
AD-RAI	10.76 (0.03, 3654.71)	0.4244	7.73 (0.02, 2723.66)	0.4942
BrainAGE	1.87 (1.21, 2.88)	0.0045	1.85 (1.20, 2.85)	0.0055
Cerebellum white matter	0.27 (0.14, 0.53)	0.0001	0.20 (0.09, 0.43)	0.0000
Cerebellum white matter	0.28 (0.14, 0.55)	0.0003	0.18 (0.07, 0.42)	0.0001
AD-RAI	56.35 (0.34, 9306.06)	0.1218	412.70 (0.73, 2.3e+05)	0.0624
Cerebellum white matter	0.29 (0.14, 0.64)	0.0018	0.19 (0.07, 0.48)	0.0004
BrainAGE	1.76 (1.19, 2.62)	0.0051	1.86 (1.15, 3.01)	0.0111
Cerebellum white matter	0.29 (0.13, 0.64)	0.0020	0.15 (0.05, 0.47)	0.0010
AD-RAI	23.04 (0.05, 10789.89)	0.3174	218.90 (0.12, 3.9e+05)	0.1577
BrainAGE	1.72 (1.15, 2.58)	0.0082	1.84 (1.13, 3.01)	0.0149

## 4. Discussion

Whether or not NPH structural changes depend strictly or not on brain aging and AD-related neurodegeneration remains a matter of intense debate. Our data in asymptomatic cases with the first MRI signs of increased risk for NPH status support a mixed scenario. They reveal that radiologically defined NPH positivity in asymptomatic elders was associated with decreased left hippocampal and cerebral and cerebellar white matter volumes that did not depend on the progression of brain aging and AD-related MRI volumetric changes supporting the idea of an idiopathic component of the disease. In other areas such as the right hippocampus, parahippocampal gyrus, and entorhinal cortex, the impact of NPH on brain volumetry seems to be strictly mediated by age-related neurodegeneration.

Early volumetric MRI studies reported increased gray matter and white matter loss in AD compared to idiopathic NPH cases in the hippocampus and perihippocampal fissures (Holodny et al., 1998). Later observations supported the idea that clinically overt NPH cases had gray matter volumes very close to controls (Serulle et al., 2014; Kojoukhova et al., 2015) or displayed minimal changes confined to the left hippocampus (Savolainen et al., 2000). Two recent studies using automatic segmentation reached different conclusions. Miskin et al. (2017) found that, in NPH cases, significant loss of gray matter occurs only in the hippocampus, whereas the gray and white matter volumes were comparable to that of age-matched healthy controls. Vlasak et al. (2021) reported significant volume loss in the left hippocampus and internal globus pallidus but also noticed overall grey and white matter in both clinically overt and suspected NPH cases. As already postulated by Golomb et al. (1994), these discrepancies may be related to the confounding effect of concomitant AD pathology in clinically overt NPH cases. The occurrence of significant AD pathology was described in approximately 30% of NPH cases (Graff-Radford and Jones, 2019), leading to the recently proposed distinction between neurodegenerative and idiopathic components of NPH (Espay et al., 2017; Muller-Schmitz et al., 2020; Vlasak et al., 2021). For some authors, dual pathology is associated with low benefits from shunting (Espay et al., 2017; Allali et al., 2018), while others defend the opposite viewpoint (Pomeraniec et al., 2016; Muller-Schmitz et al., 2020). An additional parameter to consider is the dynamics of the brain aging process that could dramatically impact on the hippocampal integrity and cognitive performances long before the emergence of incipient AD (Gaser et al., 2013; Herrmann et al., 2019).

The present study focuses on asymptomatic, radiologically defined cases at risk for NPH and takes into account automatic MRI parameters assessing the risk of AD (AD-RAI) but also the brain age gap estimation (BrainAge). By integrating these parameters in multivariable models, we were able to define whether there is a structural signature of NPH at-risk status independently of the impact of neurodegenerative changes and the brain aging process. Our data show that radiological NPH positivity is associated with a significant decrease in the volume of the left hippocampus after controlling for AD-RAI and BrainAge values. Importantly, this parameter alone explained 27.6% of the radiological NPH variance indicating that the observed association is not marginal. Our findings are consistent with previous observations (Savolainen et al., 2000; Miskin et al., 2017; Vlasak et al., 2021), pointing to the relevance of this area for the diagnosis of idiopathic forms of NPH. At first glance, the relationship between the volume of cerebral and cerebellar white matter and radiological NPH positivity may be surprising. However, several lines of evidence have indicated that both white matter microstructural changes assessed in diffusion tensor imaging studies (for review see; Horinek et al., 2016; Siasios et al., 2016; Tan et al., 2018) and deep white matter ischemia (Bradley, 2016) may be critical events in the development of clinically overt forms of NPH. Importantly, the concomitant consideration of the brain age gap and volumetric changes in white matter is the best predictor of radiological NPH status since it explains 50 to 65% of its variance.

Another set of data showed that some of the volumetric changes observed in cases at risk for NPH reflect partly the acceleration of the brain aging process. This was the case of the parahippocampal gyrus and entorhinal cortex but also right hippocampus volumes that are related to NPH scores in univariate models, yet their association did not persist when adjusting for BrainAge values. Importantly, this latter parameter alone explained more than 30% of the radiological NPH variance in models including the right hippocampus, parahippocampal gyrus, and entorhinal cortex, and its addition explained an additional 9–19% in the models including the left hippocampus and cerebral and cerebellar white matter. This finding implies that the acceleration of the brain aging process is a key player in cases at risk for NPH. In contrast, the AD-RAI values that reflect the global AD risk based on an automatic MRI volumetric assessment were not related to radiological NPH positivity at least in these asymptomatic cases. Taken together, these observations support the idea that in cases at risk for NPH, as defined by SVM assessment of NPH-related MRI markers, brain aging-related and brain aging and AD-independent volumetric changes coexist. The latter concerns volume loss in a restricted portion of the hippocampus and white matter that could be considered a possible MRI signature of idiopathic forms of NPH. It is plausible to speculate that in clinical forms of NPH in old age, and depending on the severity of concomitant AD pathology and acceleration of brain aging, the relative weight of each process changes, leading to neurodegeneration, brain aging, or idiopathic-predominant but also mixed forms of the disease. This complexity may explain the contradictory reports on the prognosis of clinically overt NPH in elderly cohorts.

Among the strengths of the present study, one should note the focus on asymptomatic cases that makes it possible to control for the confounding effect of severe AD pathology, the use of multivariable models that allows for defining the added value of each of the automatic MRI parameters, and consideration of both AD-signature and control areas in the statistical analysis. Several limitations should be considered. The small sample size limits the number of predictors that can be simultaneously included in regression models. In particular, we did not explore diffusion tensor imaging variables, which are known to be involved in the evolution of NPH, and did not control for the distinct impact of concomitant amyloid pathology. In a recent study, the latter has been used to define a hydrocephalic variant of AD (Jang et al., 2022). Moreover, the definition of radiological NPH positivity was made on the basis of an arbitrary fixed cut-off of 30. We opted for this permissive value in order to guarantee the highest sensitivity, yet it could lead to the inclusion of false positive cases. Most importantly, the main objective of our study was to identify patterns of volumetric changes in gray and white matter that were associated with radiologically defined increased risk for NPH. Subsequently, all of our cases were healthy controls without clinical evidence of NPH. We cannot thus conclude that the MRI observations made in these very early cases would not be sufficient to predict the risk of clinically overt NPH. On the basis of our observations, future studies in large samples of asymptomatic and clinically overt NPH cases, with more conservative NPH cut-offs including DTI parameters and PET amyloid data, are clearly warranted to disentangle the complex puzzle of NPH-related brain changes in old age and separate reliably the neurodegenerative, idiopathic, and mixed forms of this condition.

## Data availability statement

The raw data supporting the conclusions of this article will be made available by the authors, without undue reservation.

## Ethics statement

The studies involving humans were approved by Commission cantonale d’éthique de la recherche (CCER). The studies were conducted in accordance with the local legislation and institutional requirements. The participants provided their written informed consent to participate in this study.

## Author contributions

SH and PG conceived the study. CR and M-LM: recruitment. M-LM, CR, and PG: neuropsychology supervising. M-LM and FH: data preparation. FH, PG, and M-LM analyzed the data. PG, FH, SH, and M-LM: manuscript writing. All authors contributed to the article and approved the submitted version.

## Footnotes

^1^ http://www.neuro.uni-jena.de/cat

^2^ http://www.fil.ion.ucl.ac.uk/spm/software/spm12

^3^ www.mathworks.com

^4^ http://brain-development.org/ixi-dataset/

^5^ www.veobrain.com

^6^ https://www.cneuro.com/cmri/
